# Chimpanzees (*Pan troglodytes*) detect strange body parts: an eye-tracking study

**DOI:** 10.1007/s10071-021-01593-2

**Published:** 2022-01-28

**Authors:** Jie Gao, Ikuma Adachi, Masaki Tomonaga

**Affiliations:** 1 Primate Research Institute, Kyoto University, 41-2 Kanrin, Inuyama, Aichi 484-8506 Japan; 2 Japan Society for the Promotion of Science, Chiyoda-ku, Tokyo, 102-0083 Japan; 3 Inuyama, Aichi, 484-0000 Japan

**Keywords:** Body representation, Body structure, Chimpanzees, Eye-tracking

## Abstract

This study investigated chimpanzee body representation by testing whether chimpanzees detect strangeness in body parts. We tested six chimpanzees with edited chimpanzee body pictures in eye-tracking tasks. The target body parts were arms or legs. For either target, there were four conditions: “normal” condition as control, where all bodies were normal; “misplaced” condition, where one arm or one leg was misplaced to an incorrect body location in each picture; “replaced by a chimpanzee part” condition, where one arm or one leg was replaced by a chimpanzee leg or arm, respectively, in its original place in each picture; and “replaced by a human part” condition, where one arm or one leg was replaced by a human arm or leg in each picture. Compared to the looking times toward the normal parts, chimpanzees had significantly longer looking times toward the human arms or legs. The looking times toward the misplaced parts were also longer than the normal parts, but the difference just failed to meet significance. These results indicate more interests toward strange body parts, compared to typical parts, suggesting that chimpanzees might have a body representation that is sufficiently sensitive to detect these aspects of strangeness.

**Supplementary Information:**

The online version contains supplementary material available at 10.1007/s10071-021-01593-2.

## Introduction

A representation of typical bodies of animal species could help with species recognition, gesture and action understanding, and detection of abnormal situations such as injury. However, little research has been done in non-human animals. We examined chimpanzees’ looking behaviors to atypical chimpanzee bodies in this study aiming at providing insights about their body representation. We first introduce the potential functions of body representation and explain why we chose to test chimpanzees; then, we summarize previous research in relevant fields and reveal the paucity in this particular area; we then introduce the bases of the methodology and the study design.

All animals have bodies. They see others’ bodies frequently, and certain visual representations of typical bodies are important in their lives. For example, how do humans know other individuals are also humans? The most dominant information may come from visual cues: because they have a human body (Peelen and Downing 2007). Humans use bodily gestures to convey emotion or other social cues. The knowledge that these gestures are performed using certain human body parts is needed to understand the information conveyed: for example, the pointing gesture shows attentional goals, while other gestures or postures express various emotions (Dael et al. 2012; De Gelder 2006). In some instances, humans notice when bodies do not look typical; these situations require special attention. For example, an arm disconnected from the shoulder signifies injury and the need for medical care. All such scenarios require a visual representation of bodies, including an understanding of typical bodies and an ability to detect atypical cases. It may be important for other species as well to have a similar visual representation of their bodies.

In this study, we focused on chimpanzees, which are humans’ closest living relatives; analysis of chimpanzees could help shed light on the evolution of visual body representation (Call et al. 2017; Matsuzawa 2009). Chimpanzees have excellent visual abilities that are close to humans’, which are used extensively in their lives (Bard et al. 1995; Matsuzawa 1990; Matsuzawa et al. 2006; Spence 1934). Furthermore, chimpanzees have intensive social interactions among group members, and they must distinguish species and recognize individuals (Goodall 1986; Matsuzawa et al. 2011; Nakamura et al. 2015). They also communicate with each other using bodily produced, visual gestures (Bard et al. 2014; Hobaiter and Byrne 2011; Liebal et al. 2004). From chimpanzees’ behaviors, it is reasonable to infer that they may have a visual representation of “the chimpanzee body.” However, it is important to investigate whether there is any behavioral evidence of this visual representation.

One relevant avenue of research involves posture imitation. Chimpanzees are able to imitate humans’ natural and arbitrary body postures, including sign language, but sometimes they show difficulties in the imitation (Custance et al. 1995; Hayes and Hayes 1952; Myowa-Yamakoshi and Matsuzawa 1999). The difficulties could be from their lack of sophisticated representations of their own bodies, or from the absences of other abilities (e.g., mirroring postures from a different species or conducting behavior on the basis of their abstract body representations) because many cognitive modules and their integrations are involved in this imitation process. Therefore, imitation studies have not revealed extensive details concerning body representation in chimpanzees.

Studies about body part naming could also provide some evidence of body representation. Human children develop the ability to name human body parts at an early age. They can name commonly referenced parts, such as arms and legs, at approximately 2 years of age (Camões-Costa et al. 2011; MacWhinney et al. 1987; Mitchell 1993; Waugh and Brownell 2015; Witt et al. 1990). Among other species, dolphins can also “name” their body parts: they could represent corresponding body parts in response to gestural symbols presented by humans (Herman et al. 2001). Chimpanzees seem to be able to comprehend the names of certain body parts. For example, the chimpanzee Gua understood instructions such as “show me your nose”; she pointed at her nose after receiving this instruction. She pointed at a picture of a dog or shoes after receiving the following respective instructions: “show me the bow-wow (dog)” and “show me the shoe.” These findings suggest that she knew the meaning of “nose” (Kellogg and Kellogg 1933). However, this type of evidence is rather limited, and many other cognitive abilities are also involved in the naming process; thus, it is difficult to determine the accuracy of chimpanzee body representations from the current literature.

Studies in body visual processing also provide some indirect evidence regarding chimpanzees’ abilities to correctly represent their bodies. In humans, neurons in the extrastriate and fusiform body areas encode detailed body shapes and postures; chimpanzees also possess extrastriate areas and fusiform gyri, which implies that they might also have this body encoding system (Downing and Peelen 2011). Humans show inversion effects for bodies: they are better at recognizing bodies when they are upright than inverted (upside down). This inversion effect suggests that humans visually process bodies as a whole template and use cues of relational information between local parts, instead of focusing on the features of local parts, and this type of processing is efficient for body recognition (Reed et al. 2003). Chimpanzees also show the inversion effect for chimpanzee bodies, suggesting that they may have a body-processing mechanism similar to that of humans (Gao and Tomonaga 2018). However, this inversion effect is eliminated when body structures are scrambled. This difference in the processing of typical and atypical bodies indicates that chimpanzees may have a body representation with correct placement of body parts (Gao and Tomonaga 2020a). Furthermore, when they are shown pictures of human bodies, chimpanzees do not exhibit an inversion effect for bipedal humans, but show this effect for quadrupedal humans, despite their visual familiarity primarily with bipedal humans (Gao et al. 2020; Gao and Tomonaga 2020b). Because chimpanzees move in a quadrupedal position, these findings suggest that they may be particularly sensitive to a body structure that is similar to their own body structure, indicating a degree of conspecific body representation.

Thus far, few studies have reported comparatively direct evidence of body representation. In Hebb’s observations (1946, 1949), captive chimpanzees were either terrified or excited when they were shown a model of a human or chimpanzee head without other body parts, possibly because of the surprise involved in viewing a detached head, which violated their experience of body appearance (i.e., an intact body). In a study regarding chimpanzees’ reactions to wounds (Sato et al. 2019), chimpanzees spontaneously attended to wounds on chimpanzee bodies, suggesting that they knew the normal appearances of their body parts.

Most relevant research has either focused on a more integrative aspect of body representation or provided indirect information, and there lacks systematic examination of body representation in non-human species. In the present study, we aimed to provide direct experimental evidence of chimpanzees’ abilities to detect atypical body parts by testing their visual attention to a series of atypical chimpanzee bodies. We hoped to provide insights concerning nonhuman species’ visual representations of conspecific bodies.

Differences in looking behavior reveal different amounts of attention, thereby indicating the ability to discriminate. Looking time differences have been widely used in studies with infants and non-human animals, particularly in violation-of-expectation tasks (Gelman and Au 1996; Winters et al. 2015). For example, when presented with possible and impossible scenarios in terms of objects and gravity support, chimpanzees looked significantly longer at the impossible scenarios, suggesting that they had an awareness of the laws of gravity (Cacchione and Krist, 2004; Murai et al. 2011). Looking data have previously been obtained by video or live coding. Eye-trackers, which automatically and accurately measure the duration and location of eye gazes, have been used with increasing frequency (Aslin 2007; Kano and Tomonaga 2009; van der Geest et al. 2002). In the present study, we used eye-tracking tasks to test whether chimpanzees show different looking behaviors toward strange body parts, compared to normal body parts, to investigate their visual body representation.

We focused on arms and legs because they are prominent and major body parts. We investigated the extents to which chimpanzees knew the location and morphology (i.e., species membership) of arms and legs in pictures of unfamiliar chimpanzees. We compared chimpanzees’ attention to one body part across three experimental conditions of atypical bodies and the control condition of typical bodies. To assess chimpanzee responses to location changes, we used pictures with one arm or leg misplaced in an incorrect location. For morphology changes, we used pictures with one arm or leg replaced by a human arm or human leg at the normal body location. We also used pictures with one arm or leg replaced by another leg or arm (from the same chimpanzee body), respectively, as a combination of location and morphology cues. If chimpanzees showed shorter *time to first fixation* on the specific body part (quicker detection), or longer *fixation duration* on the specific body part (longer attention), in the experimental conditions than in the control conditions, they presumably were able to detect strangeness of arms or legs in chimpanzee bodies.

## Methods

### Participants

Six adult chimpanzees at Kyoto University Primate Research Institute (KUPRI) participated in the experiment (Table 1). They belonged to a group of 12 individuals in total. All participants were born in captivity except for Ai, who was brought to KUPRI from the wild when she was about 1 year old (details are available in the Great Ape Information Network, see Table 1). Their living environment includes an outdoor compound (700 m^2^) and attached indoor compounds. All chimpanzees had full access to food and water during the study. They engaged in cognitive tests on a daily basis, and they had experience with eye-tracking tasks (e.g., Kano and Tomonaga 2009). The daily care and use of the chimpanzees adhered to the 2010 Guidelines for the Care and Use of Laboratory Primates of KUPRI. The research proposal was approved by the Animal Welfare and Animal Care Committee of KUPRI and the Animal Research Committee of Kyoto University (#2019-064, #2020-118). All procedures adhered to the Japanese Act on the Welfare and Management of Animals.

**Table 1:** General characteristics of the six chimpanzees

Name	GAIN ID number^†^	Sex	Age (when the study started)	Kinship
Ai	0434	Female	42	Ayumu’s mother
Ayumu	0608	Male	19	Ai’s son; Pal’s paternal half sibling
Chloe	0441	Female	38	Cleo’s mother
Cleo	0609	Female	18	Chloe’s daughter
Pan	0440	Female	35	Pal’s mother
Pal	0611	Female	18	Pan’s daughter; Ayumu’s paternal half sibling

^†^Identification number for each chimpanzee listed in the database of the Great Ape Information Network (GAIN); https://shigen.nig.ac.jp/gain/

### Apparatus

**Fig. 1:**
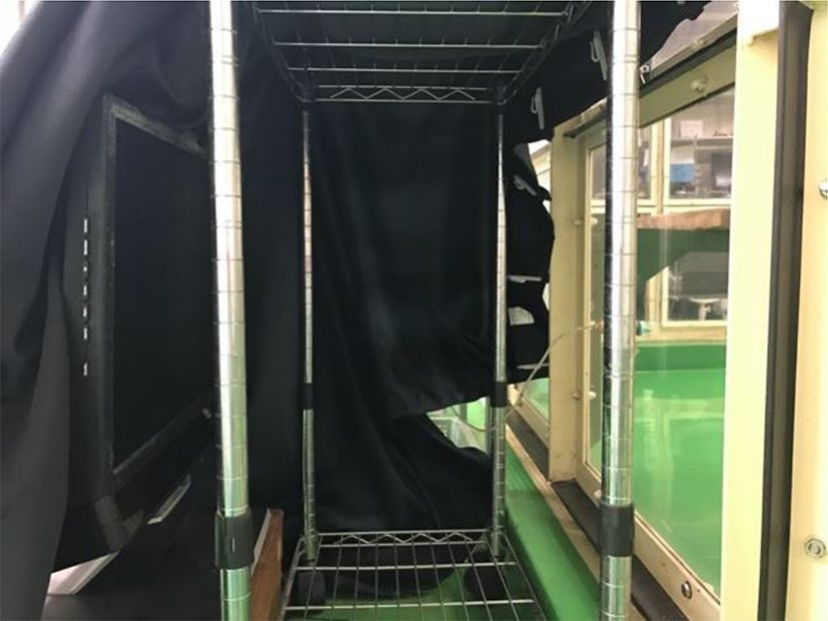
Side view of experimental setting. On the left is the display screen; the eye-tracker element is directly beneath the screen, tilted slightly away from vertical and facing the glass. The eye-tracker element was built together with the screen. In the middle of the glass on the right is a hole through which a juice tube extends. When chimpanzees drank juice from the tube, their eyes stayed at approximately the same horizontal level as the center of the screen. The setting was covered by a black cloth during the experiment

We used the Tobii TX300 Eye Tracker and Tobii Studio (Tobii Technology AB; Danderyd, Sweden) for data collection and analyses. The sampling rate of the eye tracker was 300 Hz; it had an attached 23-inch Tobii TX display (1080 × 1920 px). The screen with the eye tracker was placed in an upright position at 60 cm from chimpanzee participants (Fig. 1), and the participants viewed the screen through the glass. A tube from which the chimpanzees could drink juice during the task was inserted through a hole in the glass, allowing their heads to remain comparatively still. The height of the hole allowed chimpanzees to sit on the ground and drink juice with their eyes remaining at approximately the same horizontal level as the center of the screen. To minimize the time chimpanzees spent looking at other things, the whole setting was covered with black cloth. This did not prevent chimpanzees from looking at the cloth itself, but blocked distractions from the many colors and shapes in the experimental room.

### Stimuli

**Fig. 2:**
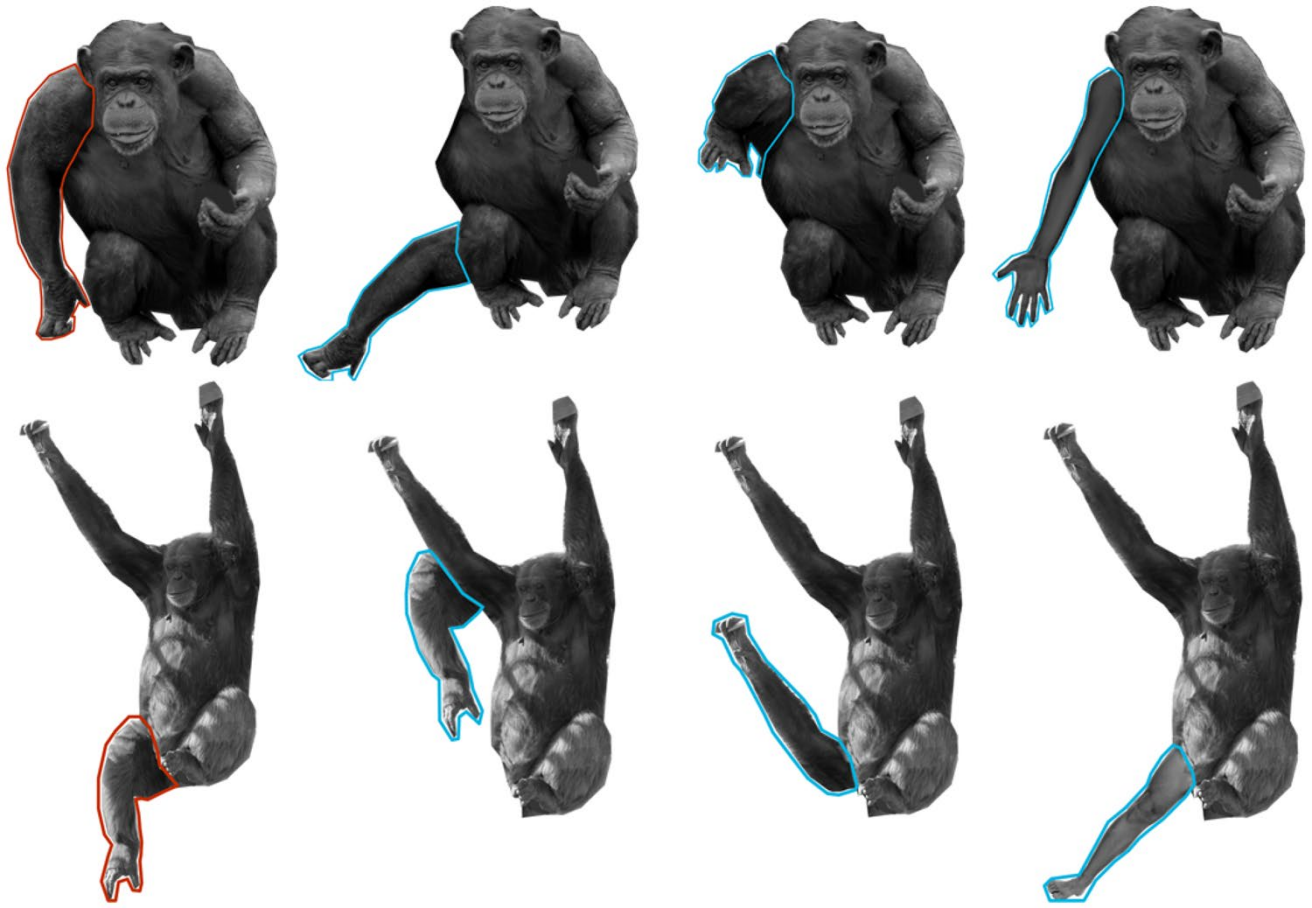
Sample stimuli and areas of interest (AOIs). The top row shows examples of the “arm” conditions; the bottom row shows examples of the “leg” conditions. In each row, from left to right, are examples of the “normal,” “misplaced,” “replaced by a chimpanzee part” (for arms, replaced by a leg; for legs, replaced by an arm), and “replaced by a human part” (for arms, replaced by a human arm; for legs, replaced by a human leg) conditions. The outlined areas with the control/manipulated body parts show the AOIs for each condition. The red areas show the normal arm or leg, and the blue areas show the manipulated results. The lines were not shown during the experiment

The targeted body part was either one arm or one leg. Regardless of the arm or leg used, there were four following conditions: “normal” (control), “misplaced,” “replaced by a chimpanzee part,” and “replaced by a human part” (Fig. 2). For arms, “replaced by a chimpanzee part” meant “replaced by a leg (of the same chimpanzee),” and “replaced by a human part” meant “replaced by a human arm.” For legs, “replaced by a chimpanzee part” meant “replaced by an arm (of the same chimpanzee),” and “replaced by a human part” meant “replaced by a human leg.” Twenty pictures were used for the control condition (“normal”). Normal or scrambled forms of the chimpanzees depicted by these pictures have appeared in previous experiments (Gao and Tomonaga 2018, 2020a, b). These experiments were conducted at least one year prior to this study and contained limited sessions over a short testing period. For each individual photograph, the body was manipulated in three ways (one “misplaced” and two “replaced”) for these three experimental conditions. Therefore, in total, 80 pictures were used to study arms, and 80 pictures were used to study legs. For example, regarding arms, all 20 stimuli under the normal condition were normal chimpanzee bodies, and these 20 pictures contained 20 chimpanzee images that were cut from 20 different chimpanzee photos. They were labeled by numbers 1, 2, 3, …, 20. For Picture No.1, one arm was cut out and then pasted on a random wrong location (where an arm should not be) of the body, and this edited picture was one of the stimuli for the “misplaced” condition; one arm was replaced with the leg of the same side, and this picture was one of the stimuli in the “replaced by a chimpanzee part (a leg)” condition; one arm was replaced with a human arm, and this picture was one of the stimuli in the “replaced by a human part (a human arm)” condition. The same was done for the other 19 pictures. Regarding legs, the manipulations were similar. Under the “misplaced” condition, one leg was cut out and then pasted on a wrong location. In the “replaced by a chimpanzee part (an arm)” condition, one arm replaced one leg. In the “replaced by a human part (a human leg)” condition, a human leg replaced one leg. The normal pictures of “arm” and “leg” conditions were not repeated. When we chose which arm or leg on the body to edit, the principle was to find the most convenient one and to keep the picture to be as “real” as possible without adding too much artificial traces. As a result, 10 left arms, 10 right arms, 5 left legs, and 15 right legs were manipulated.

All pictures were displayed on a canvas of 800 × 800 px in Pixelmator (Pixelmator Team, Ltd., Vilnius, Lithuania). We aimed to ensure that the stimuli filled the canvas, thereby ensuring that all chimpanzee bodies were of similar size. All pictures were black and white. The luminance of the manipulated parts was adjusted to fit the luminance of the surrounding area. The original pictures were provided by Kumamoto Sanctuary, Wildlife Research Center of Kyoto University.

### Procedure

We used the built-in calibration for children to calibrate the eye gaze for each chimpanzee before the experiments. Each chimpanzee typically participated in two sessions per day; occasionally, they participated in more than two sessions in a single day. Each session included four pictures (one for each of four conditions: “normal,” “misplaced,” and two “replaced”) depicting four different individuals. For example, in an “arm” session, we could have chimpanzee A (in the picture) with normal arms, chimpanzee B with a misplaced arm, chimpanzee C with one arm replaced by a leg, and chimpanzee D with one arm replaced by a human arm. In another “arm” session, we could have chimpanzee A with a misplaced arm, chimpanzee B with normal arms, chimpanzee C with one arm replaced by a human arm, and chimpanzee D with one arm replaced by a leg. Similar arrangement was made for “leg” sessions.

**Fig. 3:**
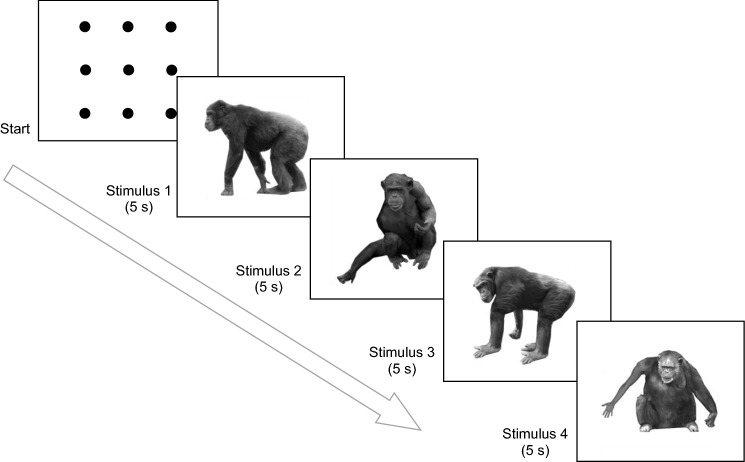
The procedure for one trial. In each session, a plot of nine dots was presented before the test stimuli to confirm calibration. When the participant’s gaze fell on one dot, the test stimuli were shown in a sequential manner, each for 5 s. The four stimuli depicted distinct chimpanzee individuals with various manipulations

A figure composed of nine dots was presented right before four stimuli in each session to confirm the calibration. Chimpanzees came to the apparatus area and sat on the ground. We provided them with juice, ensured that they were facing directly toward the display screen, and then started the task. The juice supply continued at a fixed rate throughout the session. We monitored their gaze using Tobii Studio during the task. The nine-dot figure appeared first. When the participant’s gaze fixated on one of the dots, we began to show the four stimuli (four trials) in a sequential manner. Each picture was presented in the center of the screen against a white background. Each picture was presented for 5 s and then the next one automatically appeared (Fig. 3). There was a brief break between sessions.

### Eye-tracking parameters

We used Tobii Studio’s default built-in I-VT filter with the “average” option. The window length of the velocity calculator was 20 ms. Data points with angular velocity below 30 degrees per second were classified as “fixation”; thus, any gazing sample slower than this threshold was recorded as a “fixation” (i.e., a look to the area instead of a passing-by movement from one area to another area). Typically, fixation durations were above 60 ms. The areas of interest (AOIs) were the targeted arms/legs (normal and corresponding manipulated arms/legs; Fig. 2). For example, for arms, the AOI in a “normal” picture was the arm that was manipulated in the three experimental conditions. The AOI in a “misplaced” picture was the arm that was misplaced. The AOI in a “replaced by a chimpanzee part” picture was the leg that replaced the arm (in the arm location). The AOI in a “replaced by a human part” picture was the human arm. We examined these two parameters: time to first fixation on AOIs (in each trial) and fixation duration on AOIs (in each trial).

Unlike human adults, chimpanzees do not look at the screen continuously, and they did not look at AOIs in many trials. We only analyzed data of the trials with fixations to AOIs. In each trial, time to first fixation refers to the time from the start of the trial until the chimpanzee first fixated on the AOI. In each trial, fixation duration refers to the sum of all fixation durations on the AOI. We compared these parameters among the experimental conditions and the control condition.

### Data analyses

To compare the time to first fixation and fixation duration across conditions, we used the generalized linear mixed model (GLMM) tool in R (R Core Team; Vienna, Austria 2020); the specific package was “lme4” (Bates et al. 2015). The distribution of these data is the gamma distribution. For each estimate, we tested statistical significance based on Wald's *z*-value. Regarding time to first fixation, the data included eight zero values in the “arm” category. Even for a very quick detection, the time is not likely to be below one unit of the precision of the eye-tracker to result in a zero value (Kano and Tomonaga 2011), so these zero values indicate that the participants were already looking at the target area at the onset of the stimuli. Therefore, we deleted these eight zero values in the analysis of time to first fixation. For fixation duration, we used data from all the trials with fixation duration (i.e., eight more trials than time-to-first-fixation analyses). However, it is possible that the fixation durations of these eight trials are longer than they should be, because the participants accidentally had their gaze on the AOIs at the onset of the stimuli instead of transferring their gaze to the AOIs actively. We analyzed fixation duration again without the data in these eight trials and the results could be found in the supplementary material.

The full models had fixed effects (condition [“normal,” “misplaced,” “replaced by a chimpanzee part,” or “replaced by a human part”], body part [“arm” or “leg”], and the interaction of condition and body part) and random effects (participant ID and picture ID). The null models had only random effects (participant ID and picture ID). We compared the full model and the null model first. If they differed significantly, we then examined how significant each fixed effect was. If the effect of condition or body part was significant, we then conducted post hoc pairwise comparisons based on that specific fixed effect. If there was a significant interaction between condition and body part, we conducted pairwise comparisons of all relevant pairs to examine the situation (6 comparisons for the 4 conditions of arm data, 6 comparisons for the 4 conditions of leg data, and 4 arm-leg comparisons for the 4 conditions; 16 pairs in total). For the significance level of random effects, we compared the full model and the model with one random effect dropped from the full model to examine the significance of that random effect.

## Results

### Number of trials with fixations on AOIs

The number of trials with AOI fixations under each condition is shown in Table 2. For either “arm” or “leg” sessions, there were 20 pictures in each condition, and 4 conditions and 6 participants in total, so there were 480 trials for the arm sessions (480 trials) and for the leg sessions (480 trials). Among them, the participants showed fixations in 326 trials (171 for arm manipulations and 155 for leg manipulations).

**Table 2:** Number of trials with and without fixations on AOIs under each condition

Condition	Arm manipulation		Leg manipulation	
	With fixation	Without fixation	With fixation	Without fixation
Normal	30	90	25	95
Misplaced	45	75	62	58
Replaced by a chimpanzee part	45	75	34	86
Replaced by a human part	51	69	34	86
Sum	171	309	155	325

### Time to first fixation on AOIs

**Fig. 4:**
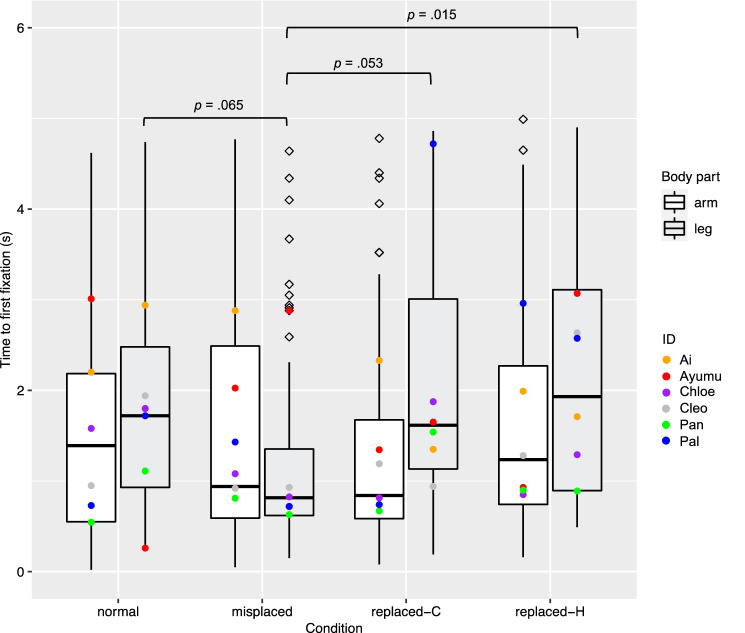
Box plots to show time to first fixation on the AOI (by trial) under each condition for manipulations of each body part. Medians in each condition are marked with a line. Hollow rhombi are outliers. Solid dots in different colors were added to show the medians of each condition by each participant. *P* values less than .07 were shown in the figure

**Fig. 5:**
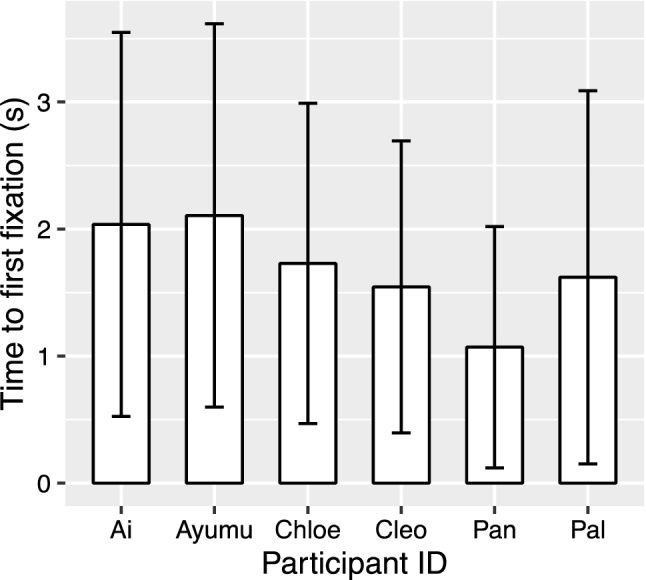
Mean time to first fixation on the AOIs of each chimpanzee participants in all conditions. Error bar: *SD*

The full model with the fixed effects being condition, body part and their interaction was significantly different from the null model, which had no fixed effects and only random effects (χ^2^(7, *N* = 6) = 15.01, *p* = 0.036). For the full model, an analysis of variance based on mixed gamma regression indicated a significant effect on time to first fixation of the interaction of condition and body part (χ^2^(3, *N* = 6) = 8.42, *p* = 0.038), but the effects of condition (χ^2^(3, *N* = 6) = 0.84, *p* = 0.84) or body part (χ^2^(1, *N* = 6) = 3.11, *p* = 0.078) were not significant (Fig. 4). Simultaneous pairwise comparisons based on either condition or body part (16 pairs in total; Table 3) indicated that for leg data, the “misplaced” condition had significantly shorter time than the “replaced by a human part” condition (*Z* = 3.26, *p* = 0.015). In the leg data, the “misplaced” condition also had shorter time to first fixation than the “normal” condition and the “replaced by a chimpanzee part” condition, but the differences just failed to meet significance (with the “normal” condition: *Z* = 2.77, *p* = 0.065; with the “replaced by a chimpanzee part” condition: *Z* = 2.85, *p* = 0.053). The comparison between the full model and the model with “participant ID” dropped from the full model showed a significant effect of the random effect participant ID (χ^2^(1, *N* = 6) = 6.19, *p* = 0.013; Fig. 5). The comparison between the full model and the model with “picture ID” dropped from the full model showed no significant effect of the random effect picture ID (χ^2^(1, *N* = 6) = 1.71, *p* = 0.19).

**Table 3:** Results of post hoc pairwise comparison of time-to-first-fixation data, based on the interaction of condition and body part

Body part	Condition	Contrast^†^	Estimate	*SE*	*Z* value	*P* value
Arm	–	Misplaced—normal	– 0.030	0.111	– 0.27	1
Arm	–	Misplaced—(replaced-C)	– 0.086	0.104	– 0.82	.97
Arm	–	Misplaced—(replaced-H)	– 0.003	0.092	– 0.03	1
Arm	–	Normal—(replaced-C)	– 0.056	0.119	– 0.47	1
Arm	–	Normal—(replaced-H)	0.027	0.109	0.25	1
Arm	–	(replaced-C)—(replaced-H)	0.083	0.102	0.81	.97
Leg	–	Misplaced—normal	0.294	0.106	2.77	.065
Leg	–	Misplaced—(replaced-C)	0.279	0.098	2.85	.053
Leg	–	Misplaced—(replaced-H)	0.313	0.096	3.26	.015
Leg	–	Normal—(replaced-C)	– 0.014	0.097	– 0.15	1
Leg	–	normal—(replaced-H)	0.019	0.095	0.20	1
Leg	–	(replaced-C)—(replaced-H)	0.034	0.085	0.40	1
–	Misplaced	Arm—leg	– 0.19	0.105	– 1.76	.51
–	Normal	Arm—leg	0.14	0.121	1.15	.87
–	Replaced-C	Arm—leg	0.18	0.107	1.69	.56
–	Replaced-H	Arm—leg	0.13	0.092	1.42	.73

^†^The comparisons are shown in the format of “item A—item B”. Each item shows the condition. “Replaced-C” is short for “replaced by a chimpanzee part”, and “replaced-H” is short for “replaced by a human part”. Each contrast has either the same body part or the same condition. For example, the data in the first line shows the contrast of “misplaced” and “normal” conditions of “arm” data

### Fixation duration on AOIs

**Fig. 6:**
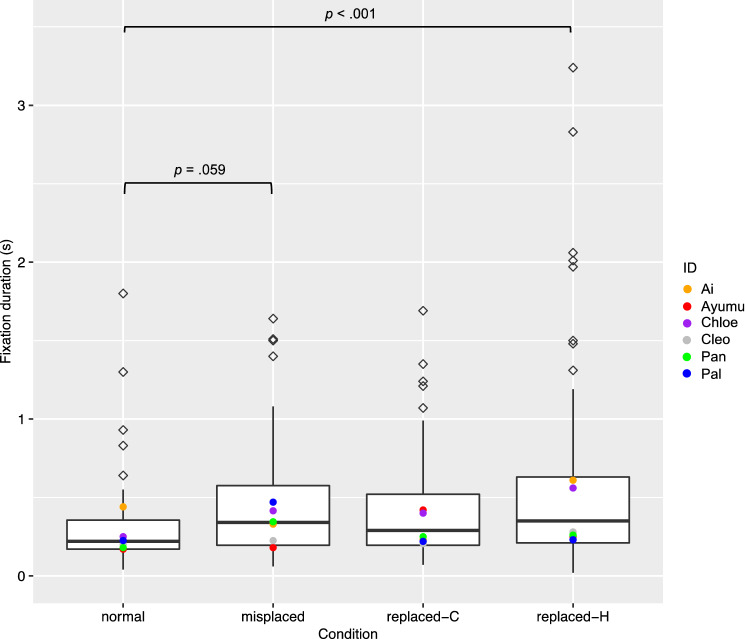
Box plots to show total fixation duration on the AOI (by trial) under each condition for manipulations of each body part. Medians in each condition are marked with a line. Hollow rhombi are outliers. Solid dots in different colors were added to show the medians of each condition by each participant. *P* values less than .07 were shown in the figure

The full model with the fixed effects being condition, body part and their interaction was significantly different from the null model, which had no fixed effects and only random effects (χ^2^(7, *N* = 6) = 24.11, *p* = 0.0011). For the full model, an analysis of variance based on mixed gamma regression indicated a significant effect of condition (χ^2^(3, *N* = 6) = 15.33, *p* = 0.0016), but the effects of body part (χ^2^(1, *N* = 6) = 0.54, *p* = 0.46) or the interaction of condition and body part (χ^2^(3, *N* = 6) = 2.95, *p* = 0.40) were not significant (Fig. 6). Simultaneous pairwise comparisons based on the effect “condition” using Tukey’s HSD test (Table 4) indicated that the “normal” condition had significantly shorter fixation duration on AOIs than the “replaced by a human part” condition (*Z* = 3.94, *p* < 0.001). The “normal” condition also had shorter fixation duration on AOIs than the “misplaced” condition, but the difference just failed to meet significance (*Z* =  – 2.51, *p* = 0.059).

**Table 4:** Results of post hoc pairwise comparison of fixation-duration data, based on condition

Contrast^†^	Estimate	*SE*	*Z* value	*P* value
Misplaced—normal	– 0.755	0.301	– 2.51	.059
Misplaced—(replaced-C)	– 0.099	0.226	– 0.44	.97
Misplaced—(replaced-H)	0.397	0.195	2.03	.18
Normal—(replaced-C)	0.655	0.314	2.09	.16
Normal—(replaced-H)	1.151	0.292	3.94	< .001
(replaced-C)—(replaced-H)	0.496	0.213	2.33	.092

^†^The comparisons are shown in the format of “item A—item B”. Each item shows the condition. “Replaced-C” is short for “replaced by a chimpanzee part”, and “replaced-H” is short for “replaced by a human part”

**Fig. 7:**
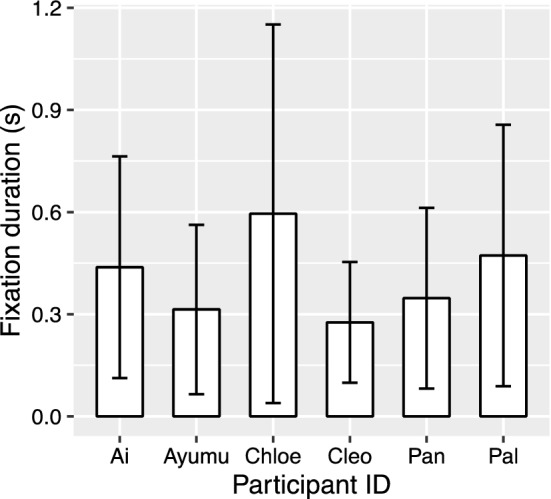
Mean fixation duration on the AOIs of each chimpanzee participants in all conditions. Error bar: *SD*

**Fig. 8:**
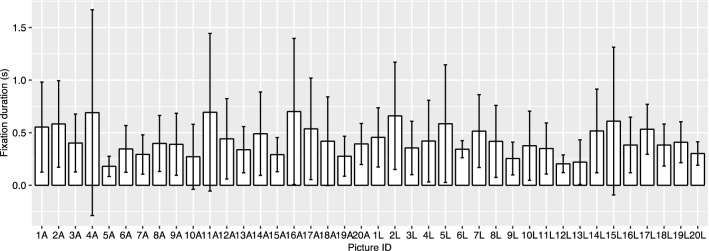
Mean fixation duration on the AOIs of each picture in all conditions. Error bar: *SD*. Regarding “picture ID”, “A” represents arm manipulations, “L” represents leg manipulations, and numbers refer to different stimulus series. For example, data points at “1A” included data the “1A” picture series: they all had the same chimpanzee individual in the pictures, but in the “normal” condition, the body was intact; in the “misplaced” condition, one arm of the body was misplaced; in the “replaced by a chimpanzee part” condition, the same arm of that body was replaced by a chimpanzee leg; and in the “replaced by a human part” condition, the same arm of that body was replaced by a human arm

The comparison between the full model and the model with “participant ID” dropped from the full model showed a significant random effect of participants ID (χ^2^(1, *N* = 6) = 46.50, *p* < 0.001; Fig. 7). The comparison between the full model and the model with “picture ID” dropped from the full model showed a significant random effect of picture ID (χ^2^(1, *N* = 6) = 18.28, *p* < 0.001; Fig. 8).

## Discussion

In this study, we tested six chimpanzees in eye-tracking tasks to examine whether they specifically attended to strange arms or legs of chimpanzee pictures, compared to normal arms or legs, to determine whether they possessed visual body representation. Compared with looking durations toward the normal body parts, the chimpanzees had significantly longer looking times toward the human arms and legs in place of the original chimpanzee arms and legs. This suggests that the chimpanzees noticed that the human parts were strange. They also showed longer looking times towards the misplaced parts than towards the normal parts, but the difference just failed to meet significance.

The “misplaced” condition and “replaced by a human part” condition showed different contrasts against the control condition: the former did not reach significance, although close, while the latter showed significance. The longest attention to the human parts is probably due to the inconsistency of the shapes, or their interests on human parts. Chimpanzees are able to detect an odd stimulus out of uniform distractors (Tomonaga 1998). Human arms and legs are hairless, and look differently from chimpanzee arms and legs, although the overall shapes are similar. In the other two experimental conditions, the manipulation was done with the body parts of the same chimpanzee body. Therefore, the special look of human body parts may have grabbed more attention. The chimpanzees we tested were very familiar with humans. They see and interact with multiple humans every day, and they could see humans in the institute and on the street. Therefore, they had been exposed to human body parts. That said, it is unlikely that they have seen the whole naked arms and legs of humans as were shown in the task; but the experience of exposure to partial human arms and legs may have triggered them to pay more attention to the human parts appearing on chimpanzee bodies and replacing chimpanzee body parts. Therefore, for the results in the “replaced by a human part” condition alone, the longer looking time could be attributed to their body representation, visual inconsistency, or interests to human bodies. To rule out the possibility that they showed longer looking time in this condition solely because of visual inconsistency or interests to humans, more control conditions could be added, or chimpanzees who have less exposure of partially naked humans could be tested. Nevertheless, when we combine all the results, we still tend to think chimpanzees may be able to detect strangeness in terms of body representation, because of the strong tendency of longer looking times towards the misplaced body parts than the normal parts and the tendency of shorter time to first fixation in the “misplaced” leg condition than the “normal” leg condition.

In the analyses of time-to-first-fixation data, we found that there was a significant interaction between condition and body part. The pairwise comparison showed significant differences in three pairs in leg data: the “misplaced” condition had shorter time to first fixation on AOIs than those in all three other conditions. It is possible that the misplaced legs make the whole body configuration look much stranger than a normal body as well as a body with its leg replaced by another part in the original typical position, leading to a much quicker detection. This was not the case for arm manipulation, and this is where the difference of the effect of condition lies for arm and leg manipulations.

The difference of results between arm and leg manipulations was not found in fixation duration, but time to first fixation, as mentioned above. In this specific case, the quicker detection to misplaced legs than legs in other conditions, but not in arms, may come from the fact that legs do not move in the same amplitude as arms. When chimpanzees move in a quadrupedal posture on the ground or in a bipedal posture when climbing, their arms and legs move in similar ranges. However, when manipulating objects on the ground, they reach for objects in places that are a bit far from them using arms not legs, and it could be seen as if the arms were “misplaced” from a distance (e.g., Hayashi and Matsuzawa 2003; Hayashi et al. 2005); chimpanzees also often raise their arms for social communications (Hobaiter and Byrne 2011), but they seldom “raise” their legs. The different function and use of arms and legs could cause chimpanzees detect misplaced legs more quickly.

In this study, we did not manipulate other body parts, such as head and torso. It will be interesting to further examine how their representation differ across various body parts. In a broader comparison counting all body parts, the difference between arms and legs may not be as large as that between head and limbs, or other contrasts. Studies asking children to recognize, name, and point at body parts do not demonstrate large differences between arms and legs, but the performances for eyes was much earlier in the development (MacWhinney et al. 1987; Waugh and Brownell 2015; Witt et al. 1990). Atypical body parts may suggest injury and care, so it is meaningful to examine whether and how knowledge for body parts differ, and which factors are related to this, such as function of the parts. Also, it will be interesting to examine the body representation of other species, too, e.g., preys. Do chimpanzees (and humans) have certain body representation and anatomy knowledge about their preys’ bodies, and do the knowledge help with efficient foraging and feeding?

All the manipulations in this study created strange images that will not occur in real life, yet the chimpanzees did not show significant differences in all manipulated conditions compared to control. One of the reasons could be due to the limited sample size. The significance of the random effect participant ID in both analyses of time-to-first-fixation data and fixation-duration data also indicates individual difference (Figs. 4, 5, 6, 7). If more individuals were tested, the results might have been more consistent. Because of the limited sample size, the conclusions should be generalized with caution, and data from more chimpanzee individuals or populations will be helpful to understand chimpanzees’ perception for atypical body parts.

The participants in this study were captive chimpanzees with a lot of exposure of humans. As discussed above, these individuals might be more sensitive to human body parts on chimpanzee bodies, compared to captive chimpanzees with limited human exposure or wild chimpanzees. However, the experience with humans may not affect chimpanzees’ body representation too much, according to our previous findings (Gao et al. 2020; Gao and Tomonaga 2020b). We tested the same chimpanzees, who were very familiar with humans, to see if they show the inversion effect for human bodies. We used humans in bipedal postures doing Tai chi, but the chimpanzees did not show any inversion effect. We then used bipedal humans showing daily postures (waving hands, walking, etc.), and the chimpanzees showed the inversion effect to these bodies, suggesting that visual experience is important to them. We also used images of crawling humans and horses in quadrupedal postures, which the chimpanzees had never seen previously, but they showed the inversion effect. Their limited inversion effects to humans, a familiar species, and the inversion effects to the quadrupedal animals that they had no visual experience about, suggest a strong tendency to refer to embodied cues, i.e., cues from their own bodies, in their body perception. Therefore, experience with humans may not affect chimpanzees’ body representation for conspecific bodies too much.

The random effect, picture ID, was significant in fixation-duration analysis (Fig. 8). This suggests that the results vary across the pictures. There are several outlier points, but not many. It is possible that the significance is related to the limited data we have: not every picture in each condition received a lot of fixations. As will be discussed below, it is inevitable to have many trials without any fixations in a chimpanzee experiment, and future studies could use more trials for more useful data points. Nevertheless, because the 20 pictures (with different kinds of manipulations) were used across conditions, this significant effect of picture ID does not interfere with the significance of condition, the main effect in the analysis.

There were several other limitations in this study. The number of trials in which the chimpanzees showed fixations to AOIs was less than half of the total trial numbers. Chimpanzees do not consistently look at the screen during a task. When they do, they typically view face and genital areas of a chimpanzee picture, while they allocate less attention to other body parts (Kano et al. 2015). More importantly, because the AOIs in this study were arms or legs (i.e., a small proportion of the whole picture), it is reasonable to have many trials without fixations on AOIs. Nevertheless, future studies in a similar setting could use a larger stimulus set to ensure more data points.

When we prepared the stimuli, the pictures were chosen randomly, and the body parts for manipulations were chosen based on picture editing convenience; we hoped to minimize editing to avoid any effects of unnatural picture manipulations. Overall, we edited 10 left arms, 10 right arms, 5 left legs, and 15 right legs. This should not fundamentally affect the experiment, because chimpanzees were in various positions (e.g., sitting, walking to the left, walking to the right, and bipedal standing), and left/right discrimination was less prominent than in a situation involving only bipedal animals. Nonetheless, future studies should carefully consider left/right bias to ensure a more balanced experimental design.

In summary, our results showed a significant longer looking time towards human body parts on chimpanzee bodies, and two non-significant tendencies: (1) shorter latencies for fixating misplaced legs, and (2) longer looking times towards misplaced parts, compared to normal body parts. These detections of strange body parts indicate that chimpanzees might have a body representation of the typical chimpanzee body. Conspecific body representation has ecological value. For example, it helps animals discriminate among conspecific individuals and individuals of other species (and then they can decide whether to fight them, socially interact with them, prey and feed on them, or ignore them). Strangeness on body parts of living individuals can indicate injury, and body representation can help trigger emotional and behavioral changes to facilitate care for these individuals (Hirata et al. 2017; Matsumoto et al. 2016; Sato et al. 2019). From an evolutionary perspective, evidence of body representation among chimpanzees indicates that the common ancestor of chimpanzees and humans might also have this type of visual representation. Of course, this conclusion needs to be supported by more data from more participants and from studies with further examinations besides arm and leg manipulations. Nevertheless, if this is true, it will lead to many interesting questions. Because both chimpanzees and humans are highly social species, and both encounter many other individuals, it is important to investigate whether body representation originates from the accumulated visual experience of conspecifics’ bodies. Investigation of this point requires examination of more solitary species, such as orangutans, as well as examination of body representation development. If the representation is present in solitary species or develops before intensive social interactions with other individuals, body representation may have more fundamental functions in animals’ life as follows: apart from aiding interactions with other individuals, body representation may be involved in many self-centered activities (Shapiro 2019). Further investigations of body representation and its interactions with other psychological processes are important for understanding how animals coordinate themselves with the outside world.

## Supplementary Information

Below is the link to the electronic supplementary material.

Supplementary file1 (PDF 264 KB)

Supplementary file2 (XLSX 28 KB)

## Data Availability

All data is available in the manuscript or the supplementary materials.

## Code availability

The codes for the experimental program and statistics are available upon request to the corresponding author.
