# Supplementary material for: Chimpanzees (Pan troglodytes) detect strange body parts: an eye-tracking study
Source: Anim Cogn. 2022 Jan 28;25(4):807–19. doi: 10.1007/s10071-021-01593-2 (PMC9334416; doi:10.1007/s10071-021-01593-2)
Supplement: Supplementary file 1 — Supplementary file1 (PDF 264 KB) [file 10071_2021_1593_MOESM1_ESM.pdf]

Chimpanzees (*Pan troglodytes*) detect strange body parts: an eye-tracking study

Jie Gao[1, 2], Ikuma Adachi[1], and Masaki Tomonaga[3]

[1]Primate Research Institute, Kyoto University, Inuyama, Aichi, 484-8506, Japan

[2]Japan Society for the Promotion of Science, Chiyoda-ku, Tokyo, 102-0083, Japan

[3]Inuyama, Aichi, 484-0000, Japan

Correspondence: Jie Gao, Primate Research Institute, Kyoto University, Kanrin 41-2, Inuyama, Aichi, 484-8506, Japan. Email: gao.jie.87c@kyoto-u.jp

Supplementary Material I

Additional data analyses of fixation duration

In eight trials of the "arm" data, the time to first fixation was zero. This suggests that the participants were already looking at the AOIs at the onset of the stimuli, which might make the fixation duration in these trials longer than they should be. Therefore, we deleted the fixation duration data from these eight trials and did the analysis again (data from 318 trials).

The full model with the fixed effects being condition, body part and their interaction was significantly different from the null model, which had no fixed effects and only random effects ($\chi^2(7, N=6) = 17.66$, $p = .014$). For the full model, an analysis of variance based on mixed gamma regression indicated a significant effect of condition ($\chi^2(3, N=6) = 9.41$, $p = .024$), but the effects of body part ($\chi^2(1, N=6) = 0.35$, $p = .56$) or the interaction of condition and body part ($\chi^2(3, N=6) = 1.81$, $p = .61$) were not

significant. Simultaneous pairwise comparisons based on the effect "condition" using Tukey's HSD test indicated that the "normal" condition had significantly shorter fixation duration on AOIs than the "replaced by a human part" condition ($Z = 3.58$, $p = .002$). Other pairs did not show significant difference (Table S1; Figure S1).

The comparison between the full model and the model with "participant ID" dropped from the full model showed a significant random effect of participants ID ($\chi^2(1, N=6) = 38.09$, $p < .001$; Figure S2). The comparison between the full model and the model with "picture ID" dropped from the full model showed a significant random effect of picture ID ($\chi^2(1, N=6) = 13.81$, $p < .001$; Figure S3).

Table S1

Results of post hoc pairwise comparison of fixation-duration data, based on condition

Contrast†	Estimate	*SE*	*Z* value	*P* value
misplaced - normal	-0.736	0.304	-2.42	.073
misplaced - replaced-C	-0.147	0.232	-0.63	.92
misplaced - replaced-H	0.332	0.202	1.65	.35
normal - replaced-C	0.589	0.319	1.84	.25
normal - replaced-H	1.068	0.298	3.58	.002
replaced-C - replaced-H	0.479	0.223	2.15	.14

† The comparisons are shown in the format of "item A - item B". Each item shows the condition. "Replaced-C" is short for "replaced by a chimpanzee part", and "replaced-H" is short for "replaced by a human part".

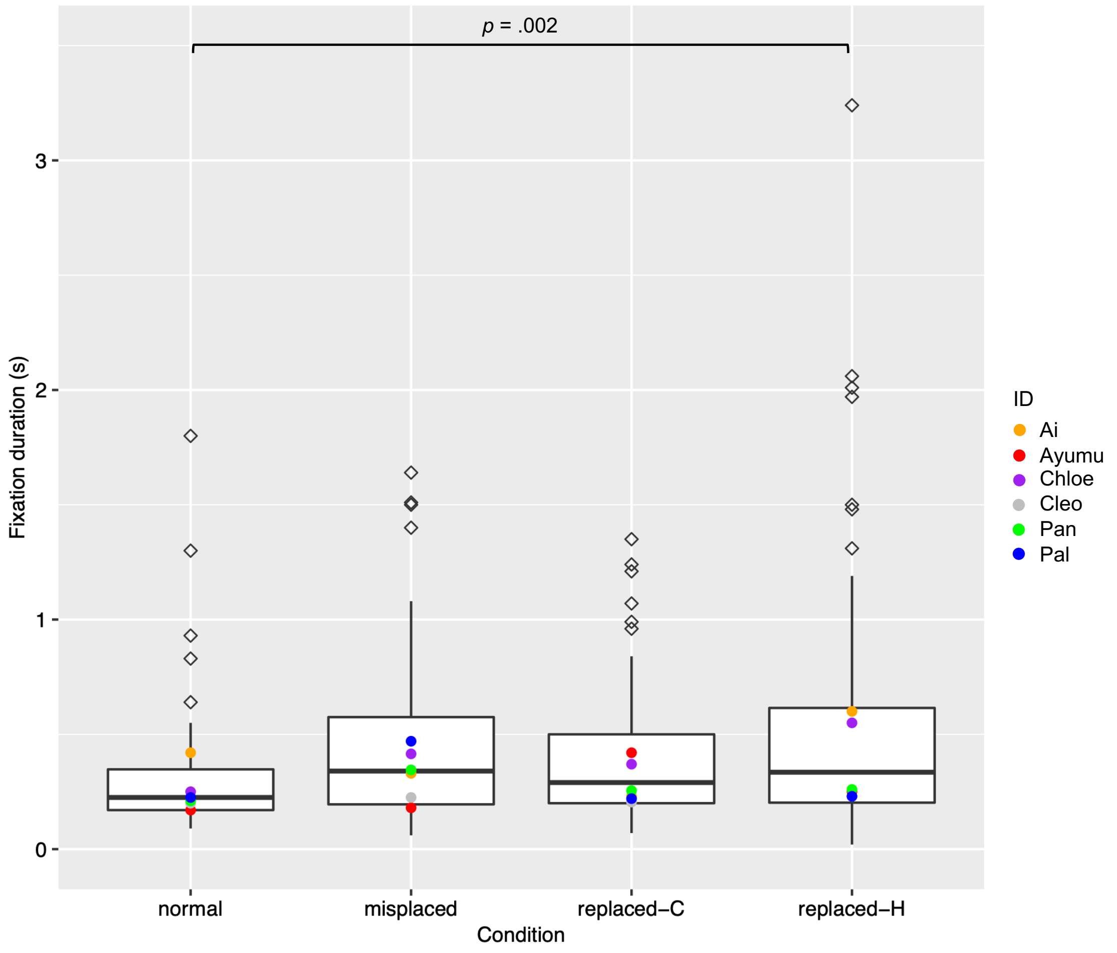

Fig. S1

Box plots to show time to first fixation on the AOI (by trial) under each condition for manipulations of each body part. Medians in each condition are marked with a line. Hollow rhombi are outliers. Solid dots in different colors were added to show the medians of each condition by each participant. The significant difference was shown in the figure with the *p* value.

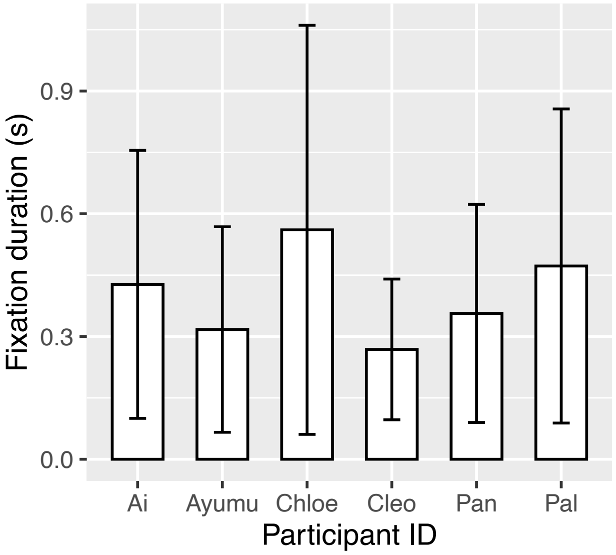

Fig. S2

Mean fixation duration on AOIs of each chimpanzee participant in all conditions. Error bar: *SD*.

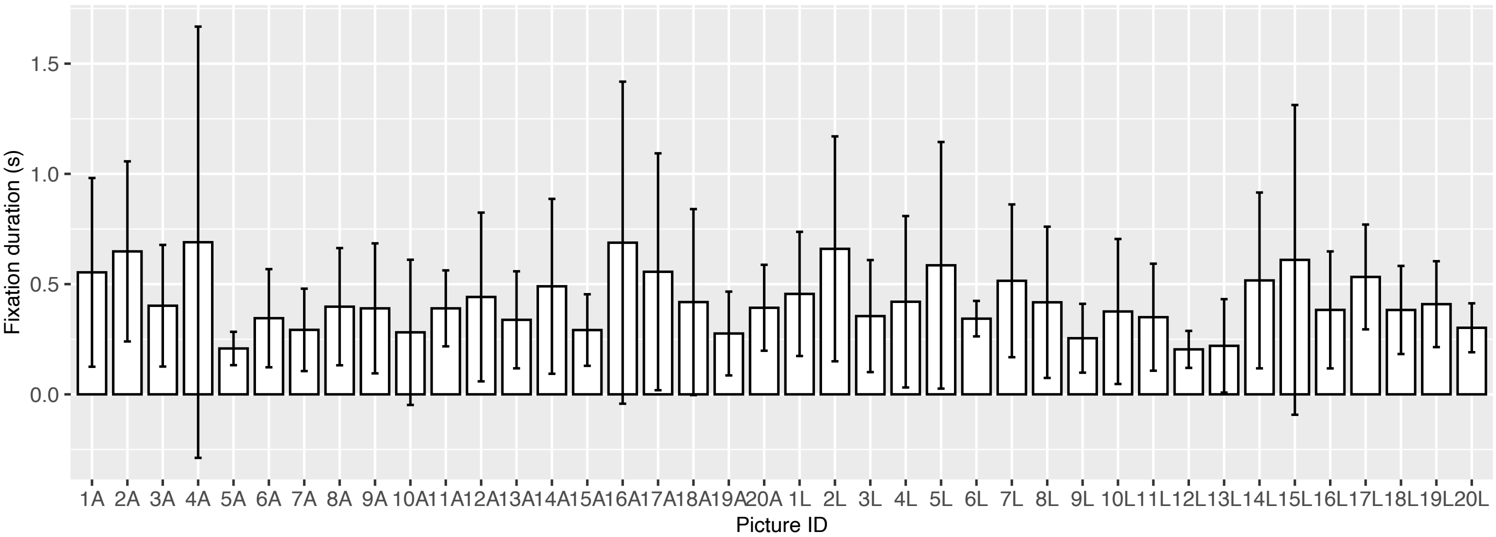

Fig. S3

Mean fixation duration on AOIs of each picture in all conditions. Error bar: *SD*.

Regarding “picture ID”, “A” represents arm manipulations, “L” represents leg

manipulations, and numbers refer to different stimulus series. For example, data at “1A” included data from the “1A” picture series: they all had the same chimpanzee individual in the pictures, but in the “normal” condition, the body was intact; in the “misplaced” condition, one arm of the body was misplaced; in the “replaced by a chimpanzee part” condition, the same arm of that body was replaced by a chimpanzee leg; and in the “replaced by a human part” condition, the same arm of that body was replaced by a human arm.
